# MRI Features in Submandibular Gland Chronic Sclerosing Sialadenitis: A Report of Three Cases and Imaging Findings

**DOI:** 10.22038/ijorl.2020.47418.2583

**Published:** 2020-11

**Authors:** Emanuela Ruberto, Emma Gangemi, Renato Covello, Raul Pellini, Antonello Vidiri

**Affiliations:** 1 *Department of Radiology and Diagnostic Imaging, IRCCS Regina Elena National Cancer Institute, Rome, Italy.*; 2 *Department of Pathology, IRCCS Regina Elena National Cancer Institute, Rome, Italy.*; 3 *Department of Otolaryngology & Head and Neck Surgery, IRCCS Regina Elena National Cancer Institute, Rome, Italy. *

**Keywords:** Chronic sclerosing sialadenitis, Head and neck, Submandibular gland

## Abstract

**Introduction:**

Chronic sclerosing sialadenitis (Küttner tumor) is a relatively uncommon and often under-recognized cause of salivary gland enlargement, characterised by sclerosing IgG4-related inflammation, producing a hard swelling of the gland that mimics malignancy. The name *tumor* is tricky and misleading, in fact the disease has no histological features of malignancy, but still it cannot easily be distinguished from cancer because of its hard consistency to touch.

**Case Reports:**

We aim to report three cases of Küttner tumor and to review morphological MRI features (homogeneous T1- and T2-hypointensity, homogeneous contrast enhancement) and diffusion weighted imaging findings (low ADC values) which can help radiologists to reach the correct diagnosis.

**Conclusion:**

Definite diagnosis of Küttner tumor is histopathological. However imaging features are straightforward and can address radiologists toward the correct diagnosis.

## Introduction

Chronic sclerosing sialadenitis is a relatively uncommon and often under-recognised cause of salivary gland enlargement, characterised by sclerosing inflammation which produces a hard swelling of the glands. Also known as Küttner tumor (named after H. Küttner, who originally described it in 1896), it has been recently recognized as a manifestation of systemic IgG4-related sclerosing disease, a new designation proposed to group lesions in different organs with similar pathological features (1). 

IgG4-related sclerosing disease has been described as a systemic disorder characterised by focal or diffuse tissue inflammation in one or more organs with characteristic histopathological findings (dense infiltrate of IgG4-positive plasma cells) associated with elevated levels of serum IgG4 (2). The name *tumor* is tricky and misleading, in fact the disease has no histological features of malignancy, but still it cannot easily be distinguished from cancer because of its hard consistency to touch (3).

The submandibular gland is more commonly affected than other salivary glands although involvement of the parotid gland and of the minor lip salivary gland has also been described (4,5). Much of the literature is based on case reports, with Küttner tumors representing in a recent paper of Sahoo et al. 4.7% of a series of 170 surgically managed salivary gland disorders and an incidence of the same setting <0.05% of all submandibular masses encountered during the period from July 2014 to January 2019 (6). Clinical examination reveals unilateral or bilateral firmness and swelling of submandibular gland, misleading clinicians towards tumor (often malignant as regards submandibular glands). Any lesion of submandibular gland mimicking a neoplasm (including Küttner tumor) should be surgically removed because tumors within the submandibular gland frequently turn to be malignant.

## Case Reports

Three middle-aged women (50,61,64 years old) presented with unilateral painless swelling and firmness of submandibular gland in the last few months. No other significant symptoms were reported. Clinical examination confirmed swelling and firmness of one submandibular gland, no adenopathies were palpable. Before presenting to our attention, two patients had undergone ultrasound examination, another a CT exam, which confirmed enlargement of the submandibular gland. Both these techniques did not yield worthwhile diagnostic clues and, in the clinical suspicion of malignancy, further investigation with MRI was required. MRI was performed with a 1.5 T scanner system (Optima MR 450w, GE Healthcare, Milwaukee, WI, USA), using a 16-channel receive-only RF coils: a head coil and surface neck. Fast spin echo (FSE) T2 –wi on the coronal plane was first obtained, followed by axial FSE T1-wi and axial FSE T2-wi with slice thickness 3 mm. Diffusion weighted imaging (DWI) was obtained by single-shot spin-echo echo planar imaging with three different b values (b = 0,500 and 800 s/mm^2^) and slice thickness of 4 mm, with all diffusion-sensitizing gradients applied in three orthogonal directions to obtain trace-weighted images. After contrast medium administration (0.1 mmol/kg), LAVA (Liver Acquisition with Volume Acceleration) sequences with slice thickness of 1 mm were performed. All MRI studies revealed increased volume of submandibular gland due to an ill-delineated submandibular lesion, characterised by an infiltrative growth pattern, showing T1 hypointensity and marked enhancement compared to the relatively unaffected parenchyma. T2 signal was low with respect to the normal gland in two out of three cases, except for one which showed T2 hyperintensity. The lesions showed restricted diffusion with high signal intensity on b 800 and low ADC values; the quantitative ADC values were obtained placing ROIs within the lesions and the values are shown in table 1.

**Table. 1:** MRI features and signal intensity of 3 cases of Küttner tumour in comparison to the contralateral gland

**CASE**	**Signal intensity on T1-wi**	**Signal intensity on T2-wi**	**Contrast Enhancement**	**Signal intensity on DWI**	**ADC value** **(x 10** ^-3^ **mm** ^2^ **/s)**
1	low	low	moderate	high	0.7
2	low	low	moderate	high	1.1
3	low	high	high	high	1.1

**Fig 1:**
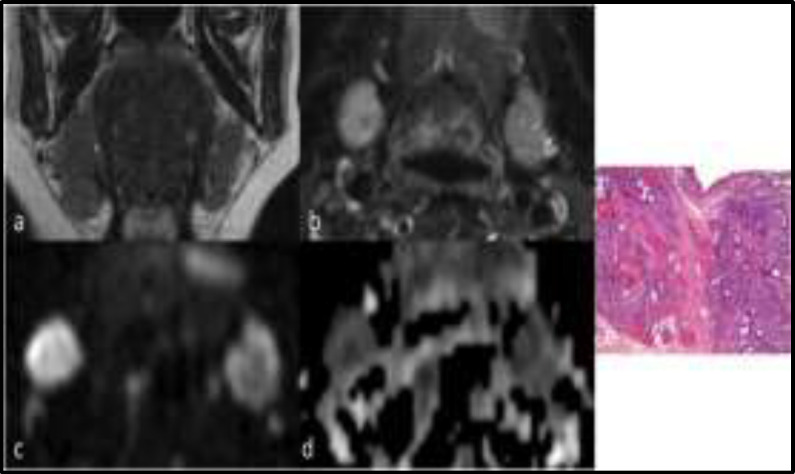
Coronal T2-wi (a)shows an enlargement of the right submandibular gland in relation to the presence of an hypointense round lesion within the inferior part of the gland; the lesion shows enhancement after contrast medium administration (b), high signal intensity on b 1000 diffusion images (c) and low values on ADC map (d) -ADC of 1.1 x10^-3^ mm^2^/s- ; the pathological specimen (e) shows extensive fibrosis, mild to moderate chronic inflammation, acinar atrophy and dilatation of some ducts. (H&E original magnification 50X). The lesion was a Küttner tumor

**Fig 2:**
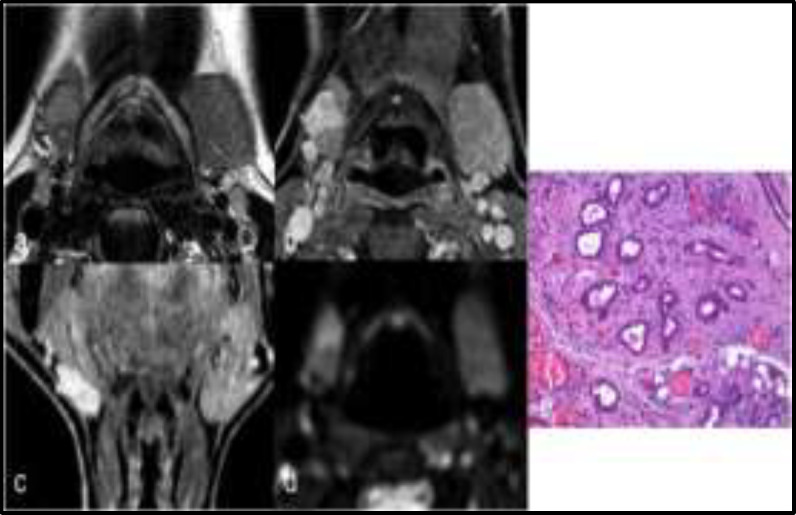
Axial T2-wi shows a lesion within the right submandibular gland, slightly hyperintense (a); the lesion shows enhancement after contrast medium administration (b, c), high signal intensity on b 1000 diffusion images (d) with ADC value of 1.1 x10-3 mm2/s; the pathological specimen (e) shows that lobular pattern is preserved even if it is distorted by fibrotic tissue. (H&E original magnification 100X). The lesion was a Küttner  tumor

**Fig 3:**
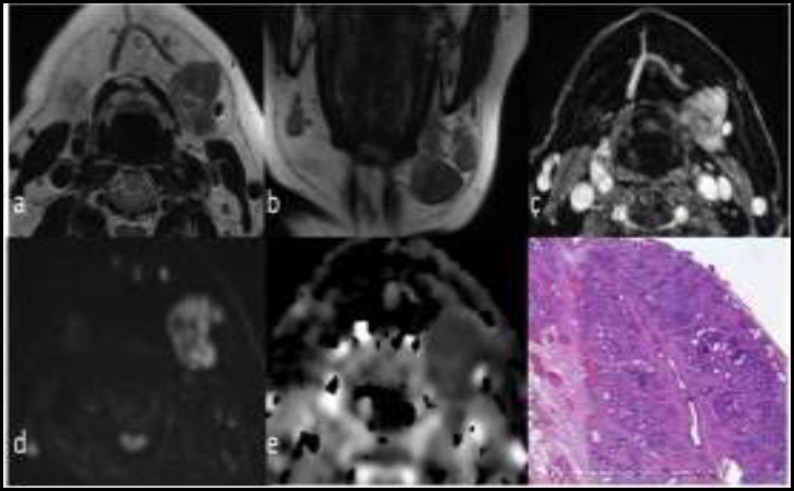
Axial (a) and coronal (b) T2-wi show a lesion in the left submandibular gland with low signal intensity, homogeneous enhancement after contrast medium administration (c), high signal intensity on b 1000 diffusion images (d) with low values of ADC -0.7 x10-3 mm2/s- (e); the pathological specimen (f) shows that lobular pattern is preserved although it is distorted by fibrotic tissue and chronic inflammation (H&E original magnification 50X). The lesion was a Küttner tumor

 Excisional biopsy was performed to exclude malignancy. Histopathological examination showed a yellowish lesion within submandibular gland characterised by a fibrotic inflammatory process with destruction of lobular architecture and acinar atrophy, associated with massive lymphatic follicular hyperplasia and massive plasmacytic infiltration, compatible with chronic sclerosing sialadenitis (Küttner tumor, Fig.1, Fig.2, Fig.3). 

## Discussion

Although chronic sclerosing sialadenitis is a rare condition, interest in Küttner tumor has increased as it has become part of a broader, often multiorgan, disease (IgG4-related), whose pathophysiology has not been fully understood.

Histopathologists are on the front line in the diagnosis of Küttner tumor; however radiologists should be able to recognize this condition and to include this entity in the range of differential diagnosis when approaching submandibular lesions. 

Classical histological findings are lymphoplasmacytic infiltrates and extensive fibrosis, associated with presence of abundant IgG4-positive plasma cells (7). Four progressive stages have been described: lymphocytic infiltration around the salivary ducts (stage 1); diffuse lymphocytic infiltration and severe periductal fibrosis (stage 2); prominent lymphocytic infiltration, parenchymal atrophy and periductal sclerosis (stage 3); marked parenchymal loss and sclerosis (stage 4) (8). However, the state of the art knowledge about Küttner tumor focuses on histological finding of IgG4-positive plasma cells infiltration. This particular finding allows chronic sclerosing sialadenitis to be distinguished from aspecific chronic sialadenitis and to be included in a systemic disorder named IgG4-related systemic disease. IgG4-related systemic disease was described for the first time in autoimmune (sclerosing) pancreatitis, characterised by diffuse lymphoplasmacytic infiltrates, presence of abundant IgG4-positive plasma cells, and extensive fibrosis (9). Since then, many extrapancreatic manifestations have been reported and these histopathological hallmarks were found in different organs involved either in the head and neck (salivary and lacrimal glands, orbits, lymph nodes, thyroid, sino-nasal-cavities, pituitary gland) and in other districts (lymphadenopathy, aortitis, sclerosing cholangitis, tubulointerstitial nephritis, retroperitoneal fibrosis). In the head and neck, some pathologic entities such as orbital pseudotumor, Mikulicz disease, Küttner tumor, and Hashimoto and Riedel thyroiditis have been regarded as part of a more complex systemic disease (10,11). Multiorgan involvement can occur synchronously or metachronously in a single patient, and the disease usually presents after 50 years of age (12). Recent studies have tried to deepen the knowledge of pathogenesis in IgG4-related disease, highlighting the critical role of interactions among clonally expanded B cells and a novel population of effector memory CD4+ T cells with a cytotoxic function (CD4+ CTLs) found out in affected tissues (13). 

As regards imaging features, in the literature there are only a few papers describing radiological findings of Küttner tumors, case reports describing prevalently US and CT features (14,15), few about MRI findings (16), a paper from Abu et al. regarding MRI of chronic sclerosing sialoadenitis (17) and a review of Radiographics about IgG4-related disease of the head and neck (18). In some cases regional lymph nodes involvement has been reported (16,19). Despite its poor specificity, ultrasonography (US) is usually the first technique of choice in the evaluation of submandibular gland enlargement; Küttner tumors are typically hypoechoic and variable patterns have been reported (reticular/nodal pattern, irregular, net- like appearance, heterogeneous echotexture, diffuse hypoechogenicity), overlapping with Sjogren’s syndrome features (14). When US is performed, fine needle aspiration citology (FNAC) can be added to allow management of submandibular gland lesions, though discrepancy between cytological and final histological diagnosis are frequent. Numerous lymphoid cells can be found in Kuttner tumor hindering the diagnosis of lymphoma (19) or chronic sclerosing sialadenitis can be not detected on FNAC but subsequently histologically proven (15). Generally in case reports found in the literature FNAC showed non specific inflammation. CT generally is not the appropriate tool to study salivary glands –apart from a few indications such as sialolithiasis and abscesses. In case of Küttner tumors findings are very aspecific, depicting areas of homogeneous attenuation within an enlarged submandibular gland. MRI is often required after a first ultrasound performance for a more elaborate evaluation of salivary gland lesions. MRI findings in Küttner tumor reported in the literature, in agreement with our cases, are consistent with infiltrative growth pattern/sometimes tumor-like mass, characterised by T1 and T2 homogeneous hypointensity (though T2 hyperintensity has also been reported), diffuse and homogeneous contrast enhancement and restricted diffusion corresponding to low ADC values (17). In our cases ADC values do not show significant differences between Küttner tumors and malignant tumors, in accord with the work of Abu A. et al (17), but there are differences with pleomorphic adenomas (typically characterised by high ADC values, ADC>1.3 x10^-3^ mm^2^/s) and lymphoma (generally characterised by low ADC value, ADC < 0.77x 10^-3^ mm^2^/s) (20). In one case of our series ADC values were similar to those expected in lymphomas; pathological examination of the lesion showed fibrotic tissue and chronic inflammation. 

## Conclusion

Küttner tumor is a rare benign disease of submandibular gland, mono or bilateral, whose clinical features simulate a submandibular tumor. Though histopathological examination is necessary for final diagnosis, in the presence of a submandibular mass MRI can be helpful in the differential diagnosis with pleomorphic adenomas and other malignant salivary tumors. Moreover MRI allows to make a reliable hypothesis of Küttner tumor when typical features such as homogeneous signal, T1- and T2-hypointensity, homogeneous contrast enhancement and diffusion restriction (low ADC values) are present. Our cases confirm MRI findings reported in the literature, providing a complete iconography of either MR sequences or histological pictures, often lacking in other papers on the subject.
